# Characterisation of p53 status at the gene, chromosomal and protein levels in oesophageal adenocarcinoma

**DOI:** 10.1038/sj.bjc.6601323

**Published:** 2003-10-28

**Authors:** S H Doak, G J S Jenkins, E M Parry, A P Griffiths, V Shah, J N Baxter, J M Parry

**Affiliations:** 1 Human Molecular Pathology Group, School of Biological Sciences, University of Wales, Swansea SA2 8PP, UK; 2 Department of Pathology, Morriston Hospital, Swansea SA6 6NL, UK; 3 Department of Pathology, Singleton Hospital, Swansea SA2 8QA, UK; 4 Department of Surgery, Morriston Hospital, Swansea SA6 6NL, UK

**Keywords:** oesophageal adenocarcinoma, p53, mutations, deletion, expression, immunohistochemistry

## Abstract

p53 mutations and loss of heterozygosity have been commonly associated with oesophageal adenocarcinoma. In this investigation, the p53 status of a Welsh population of Barrett's-associated oesophageal adenocarcinomas were fully characterised at the gene sequence, chromosomal, mRNA and protein levels. In total, 31 tumours were examined for p53 gene sequence mutations using RFLP with sequencing, allelic loss of the gene was characterised by FISH, mRNA expression by p53 pathway signalling arrays and protein levels by p53 immunohistochemistry. In all, 9.6% of adenocarcinomas harboured p53 mutations, 24% displayed p53 allelic loss and 83% exhibited p53 protein accumulation. Point mutations and deletions of the gene did not coexist within the same samples. All samples containing p53 mutations also displayed positive immunostaining; however; in the majority of cases, p53 protein accumulation developed in the absence of mutations. The gene expression analysis demonstrated no differences in p53 and mdm-2 transcription levels between the p53 immunonegative and immunopositive samples, indicating other mechanisms underlie the proteins' overexpression. In conclusion, p53 mutations and deletions do not appear to be frequent events in oesophageal adenocarcinomas; however, abnormal accumulation of the protein is present in a vast majority of cases. P53 gene mutations are not the primary cause of protein overexpression – an alternative mechanism is responsible for the positive p53 immunohistochemistry detected.

The incidence of oesophageal adenocarcinoma has risen at a greater rate than any other malignancy in the USA and most European regions, currently varying from one in 146 to one in 285 cases/patient/year (Drewitz *et al*, 1997; O'Connor *et al*, 1999; Botterweck *et al*, 2000; Nilsson *et al*, 2000; Conio *et al*, 2001). Adenocarcinoma of the oesophagus almost always arises as a consequence of the neoplastic progression of Barrett's oesophagus and has a poor prognosis, with 5-year survival rates of only 10% (Farrow and Vaughan, 1996). It is seldom found early enough for curative treatment, thus 93% of diagnosed patients die due to early vascular invasion and metastasis, making this a very aggressive malignancy (Neshat *et al*, 1994).

Barrett's oesophagus and its associated oesophageal adenocarcinoma show p53 alterations like many other malignant conditions, with allelic loss and mutations being the most commonly documented means of gene inactivation. Loss of heterozygosity (LOH) of the p53 locus has been found in 75–80% of oesophageal adenocarcinomas (Gleeson *et al*, 1998; Morgan *et al*, 1998; Wu *et al*, 1998), as well as in 79% of high-grade dysplasia (HGD), 42% of low-grade dysplasia (LGD) and in 14% of Barrett's metaplastic tissue (Wu *et al*, 1998). Hence, suggesting LOH of the p53 locus is an important change that develops early during Barrett's neoplastic progression.

p53 mutations frequently arise in both Barrett's oesophagus and oesophageal adenocarcinoma, occurring in 40–88% of cancers (Hamelin *et al*, 1994; Gleeson *et al*, 1995, 1998; Soslow *et al*, 1999) and 30–66% of Barrett's epithelium with mild or no dysplasia (Hamelin *et al*, 1994; Neshat *et al*, 1994). Between 50 and 80% of documented Barrett's p53 mutations are mis-sense (GC → AT base transitions at CpG islands) and 10–50% are nonsense (Huang *et al*, 1993; Gleeson *et al*, 1998). In both HGD and adenocarcinoma, mutations have been found between codons 152–306, but the most common alterations are transitions, 53% of which occur at the CpG dinucleotide hotspot codons 175, 196, 213, 245, 248, 273 and 282 (www.iarc.fr). Mutations at these sites disrupt p53s ability to bind DNA and regulate stress response genes. The result is an accumulation of errors in the genome, which is passed onto daughter cells thus aiding neoplastic progression. This may explain why Barrett's patients with p53 mutations have more advanced tumours, significantly worse prognosis and are on average 15 years younger than those without such alterations (Ireland *et al*, 2000). As these mutated p53 molecules have an altered conformation and can no longer form a complex with Mdm2, they are not degraded. P53 protein accumulation has been detected by immunohistochemistry in 7% of LGD, 30–60% of HGD and 45–85% of adenocarcinoma (with most reports closer to the 80% level), but not in metaplastic Barrett's epithelia (Moskaluk *et al*, 1996; Coggi *et al*, 1997; Rioux-Leclercq *et al*, 1999).

Mutated p53 can also act in a dominant-negative fashion to inactivate wild-type p53 molecules, but this is not the case for all p53 mutations and a weak p53 response can still prevail when only one allele is defective. Complete loss of wild-type p53 protein within a cell rarely occurs as a result of homozygous deletions or a double mutation, the second allele is usually inactivated by an alternate mechanism to the first. When Barrett's metaplastic tissue was examined, it was found that 95–100% of cases with 17p LOH also had p53 gene mutations (Gleeson *et al*, 1998; Barrett *et al*, 1999), while patients without LOH of the gene still carried a mutated p53 allele (Dolan *et al*, 1999). This suggests a mutation in one allele of the p53 gene probably occurs first followed by allelic loss of the second during neoplastic progression, resulting in a p53 null phenotype that can promote tumorigenesis in Barrett's oesophagus.

Indirect inactivation of p53 in oesophageal adenocarcinoma also occurs as a result of mdm2 overexpression, which has been found in 55% of adenocarcinomas (Soslow *et al*, 1999). The primary function of mdm2 is inhibition of p53 activity by directly interacting with its specific DNA binding/transactivation sites (Ashcroft and Vousden, 1999). Although mdm2 is also involved in promoting p53 degradation by transporting it from the nucleus to cytoplasm, its overexpression in human tumours maintains endogenous p53 in a nonfunctional state (Chen *et al*, 1996). It is therefore interesting to note that mdm2 overexpression in oesophageal adenocarcinomas only occurs in patients without p53 mutations (Soslow *et al*, 1999). Hence, the resultant stabilisation of wild-type p53 protein may be an alternative mechanism for loss of its protective functioning.

In this study, the p53 status in a Welsh population of oesophageal adenocarcinomas was characterised at both the gene and protein levels by looking for gene sequence mutations, allelic deletions, gene expression alterations and accumulation of the p53 protein. Examination of the interactions between the differing p53 alterations investigated provide an insight into the frequency and underlying mechanisms of p53 inactivation that are selected for during Barrett's associated neoplastic progression.

## MATERIALS AND METHODS

### Patient samples

In all, 31 cases of Barrett's adenocarcinoma were retrieved from the archives of the Departments of Pathology in Singleton and Morriston Hospitals (Swansea, UK). The male : female ratio was 4 : 1 with an age range of 42–95 years (median of 66.5 years). The samples had been formalin-fixed then paraffin-embedded and included both biopsies and surgical resections. In total 8 × 4 *μ*m and 1 × 20 *μ*m sections per patient sample were cut:

- 1 × 4 *μ*m section was stained with haematoxylin & eosin to facilitate accurate identification of the malignant regions within the sections (by APG and VS).
- 5 × 4 *μ*m sections on glass slides were used for DNA extraction (gene mutation analysis).
- 1 × 4 *μ*m section on an Apes slide was used for investigation using fluorescence *in situ* hybridisation (FISH; for gene dosage examination).
- 1 × 4 *μ*m section also on an Apes slide was for immunohistochemical analysis (protein level investigation).
- 1 × 20 *μ*m section on a glass slide was required for total RNA extraction (gene expression analysis).

### Mutation analysis

To enrich for tumour cells, DNA was only extracted from the specific neoplastic areas within the 5 × 4 *μ*m sections/patient. The samples were dewaxed and the DNA extraction was performed utilising a Stratagene Kit (Amsterdam, The Netherlands).

As the final concentration of DNA extracted was low, nested PCR was used to amplify p53 exons 5–8 from each sample (Kusser *et al*, 1993; Yoshida *et al*, 2003). The whole region between exons 5 and 8 (inclusive) was amplified initially and then subsequently used as the template for amplification of each individual exon. Table 1

**Table 1:** Primer sequences and their respective annealing temperatures for both p53 amplification and nucleotide sequencing

**Region for amplification**	**Primer annealing temperature (°C)**	**Forward primer sequence**	**Reverse primer sequence**
Exons 5–8	59	CACTTGTGCCCTGACTTTCAAC	AAAAGTGAATCTGAGGCATAAC
Exon 5	60	CCGCGCCATGGCCATCT	GCGCTCATGGTGGGGG
Exon 6	65	GTCCCCAGGCCTCTGATTCCTC	TAACCCCTCCTCCCAGAGACCCCAG
Exon 7	60	CTTGCCACAGGTCTCCCCAA	AGGGGTCAGCGGCAAGCAGA
Outer exon 8	60	GGACAGGTAGGACCTGATTTCC	AAAAGTGAATCTGAGGCATAAC
Inner exon 8	60	ACTGCCTCTTGCTTCTCTTTTCCTATCC	CTTGGTCTCCTCCACCGCTTCTTG

details the PCR primers and their respective annealing temperatures utilised with standard PCR procedures (Jenkins *et al*, 2003; Yoshida *et al*, 2003).

Restriction fragment length polymorphism (RFLP) was employed to rapidly screen for mutations within p53 hotspot codons. The normal sequence at codons 175 (exon 5), 213 (exon 6) and both 248 (exon 7) and 282 (exon 8) were digested with *Hha*1, *Taq*1 and *Msp*1, respectively. Bst 71I & Hpy CH4V digested GGC to AGC & CGT to CAT mutants at codons 245 (exon 7) and 273 (exon 8), respectively. To characterise hotspot mutations detected by RFLP and identify mutations that lay outside the codons examined, the p53 exons 5–8 from each sample were sequenced in both forward and reverse directions. The primers used for sequencing are detailed in Table 1 and any mutation detected was verified by sequencing a second PCR product to rule out artefacts introduced by Taq error.

### Immunohistochemistry

One paraffin-embedded section from each of the 31 oesophageal adenocarcinomas was processed, according to the standard ABC immunoperoxidase procedure for p53 immunohistochemical analysis and using mouse anti-human monoclonal antibody DO-7 (Novocastra Laboratories, Peterborough, UK). The antibody was diluted 1 : 50 in PBS and was applied as previously described (Freedman and Maddox, 2001). The immunostaining intensity was graded as: 0, immunonegative; 1, weak; 2, moderate; 3, strong positivity.

### FISH

Slides were dewaxed in three 10-min xylene washes, dehydrated in two 5-min 100% ethanol washes and then allowed to air dry. Tissue sections were permeabilised using a Paraffin Pre-treatment Reagent Kit (Vysis, Surrey, UK) according to the manufacturers' instructions; however, the protease solution digestion was extended to 25 min.

FISH probes LSI p53 (orange) and CEP 8 (green) (Vysis; Surrey, UK) were subsequently cohybridised onto to the pretreated sections from each sample as previously described (Doak *et al*, 2003). In all, 200 nuclei/slide were scored for the loss or gain of p53 and chromosome 8 signals. Nuclei that were obviously damaged or overlapping were excluded from the analysis, and as two probes were used in the hybridisation, nuclei containing a single signal from both probes were also omitted to minimise error caused primarily by signal truncation due to the sectioning procedure. To establish the background hybridisation variation, the probes were applied to and scored on five control sections consisting of normal squamous oesophageal epithelium. The cutoff levels for definition of deletions/amplifications were defined as the mean percentage of cells +3 s.d. of the signal losses/gains displayed in the control samples evaluated (Debiec-Rychter *et al*, 2001; Doak *et al*, 2003). No amplification of either probe was seen in the controls, but signal losses were found in 4.5–9.5% and 5.5–10.5% of cells scored per control sample for CEP 8 and LSI p53, respectively. Consequently, signal losses had to be present in more than 13.9 and 13.7% of cells for CEP8 and LSI p53 respectively to be considered a significant deletion.

### p53 pathway gene expression patterns

The total RNA was extracted from 2 × fresh biopsies taken within an area of normal squamous cell epithelium from 3 patients using the TRIspin method (Reno *et al*, 1997). The Paraffin Block RNA Isolation Kit (Ambion, Cambridgshire, UK) was also used to extract the total RNA from 10 × 20 *μ*m paraffin-embedded oesophageal adenocarcinoma sections. The archival samples selected originated from all five of the patients that had p53 immunonegative tumours, and the five that displayed the strongest p53 protein accumulation (in the immunohistochemical analysis). The isolated RNAs from the immunopositive samples were pooled together, as were the RNA extracted from the five immunonegative tissues, to collect a sufficient quantity for the subsequent gene expression analysis. Pooling the samples increased their total volume to 50 *μ*l; hence, to further concentrate them, the RNA was precipitated and resuspended in 10 *μ*l sterile RNase-free water.

The three RNA samples (immunopositive and immunonegative adenocarcinomas and normal squamous) were applied to the GEArray Q Series Human p53 Signalling Pathway Gene Array (SuperArray, Cambridge, UK), according to the manufacturers' instructions. A total of 96 genes involved in p53 upstream and downstream signalling, in addition to the p53 family itself, were represented on the arrays utilised.

## RESULTS

The integrated results for the p53 mutation, deletion and immunohistochemical analyses are detailed in Table 2

**Table 2:** p53 status in oesophageal adenocarcinoma

	**Mutations in p53**					
**Sample**	**Exon**	**Codon**	**Base change**	**Amino-acid substitution**	**P53 IHC^a^**	**p53 deletion (% of cells)**
1					+	
2					+++	
3					0	
4					+++	
5					+++	
6					+++	
7	7	245	GGC → GAC	Gly → Asp	++	
	7	247	AAC → AAT	Asn → Asn		
8					NA	NA
9					++	
10					+++	
11					+++	
12					+++	
13					++	17.5
14					+++	14.5
15					+++	24.0
16					0	
17					0	
18					0	21.5
19					+++	
20					+++	14.0
21	7	249	AGG → GGG	Arg → Gly	+++	
22					+++	
23					+	14.0
24					+++	
26					+++	
28					0	14.0
29	6	214	CAT → CGT	His → Arg	+++	
30					+++	
31					+++	
32					+++	
33					NA	NA

NA indicates samples that were unavailable for the immunohistochemical and FISH analyses; all blank cells indicate no p53 mutations or deletions (above the cutoff percentage) were found in the samples.

a p53 immunohistochemistry: 0=immuno-negative; +=weak; ++=moderate; +++=strong p53 staining.

, thus demonstrating the p53 status in each oesophageal adenocarcinoma examined.

### p53 mutation analysis

Mutations in the p53 gene were found in 9.6% (3/31) of oesophageal adenocarcinomas. Four base substitutions were identified in total (listed in Table 2): samples 21 and 29 harboured mutations at codons 249 and 214, respectively, while sample 7 contained two mutations, one at codon 245 and a second silent mutation at codon 247. Three mutations were found in exon 7 and one in exon 6 with all involving a base transition. Of the nonsynonymous mis-sense mutations, two were an A → G transition and the third was G → A at a CpG site.

### p53 allelic loss

**Figure 1:**
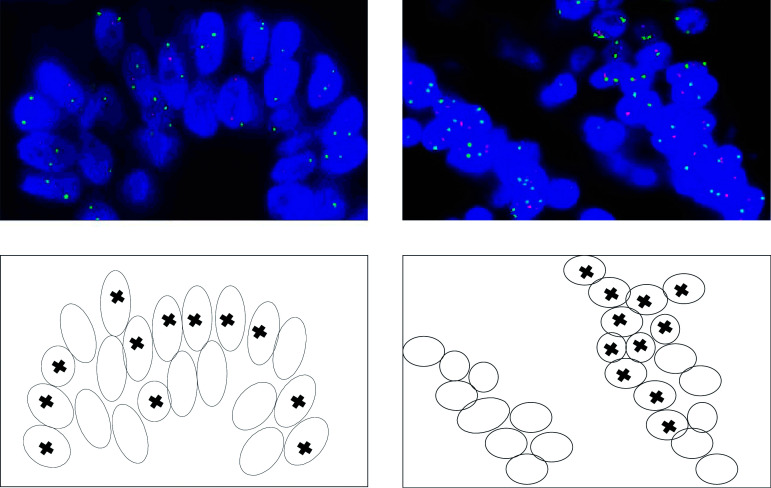
FISH images displaying p53 (red probe) deletions in a cluster of cells within an oesophageal adenocarcinoma. The green signal represents the centromeric region of chromosome 8. Coupled maps of the images indicate the cells demonstrating loss of a single copy of p53.

The loss of one p53 allele detected by FISH at a level higher than the defined cutoff was found in 24% (7/29) of oesophageal adenocarcinomas. The proportion of cells within each sample containing this aberration ranged from 14 to 24% (Table 2). The cells bearing p53 deletions were generally clustered together (as illustrated in Figure 1

) rather than scattered throughout the tumour, suggesting clonal expansion.

### Immunohistochemical p53 overexpression

Accumulation of the p53 protein was detected in 83% (24/29) of the oesophageal adenocarcinomas examined. Weak-to-moderate nuclear p53 immunostaining was observed in five samples, while the remaining 19 samples all had strong immunostaining for the protein (Table 2).

### Relationship between P53 mutation, deletion and protein overexpression in oesophageal adenocarcinoma

Immunopositivity for the p53 protein was detected far more frequently than mutation or deletion of the gene. Only three out of 24 cases immunopositive for p53 contained a mutated gene. Two out of the three samples with mutations exhibited strong p53 staining, but the sample harbouring two mutations in the p53 gene (number 7) only scored moderately for p53 protein accumulation.

Oesophageal adenocarcinomas with p53 mutations did not contain gene deletions in addition. Notably, allelic loss of p53 was associated with 40% (two out of five) of the p53 immunonegative samples.

### p53 pathway gene expression patterns

**Figure 2:**
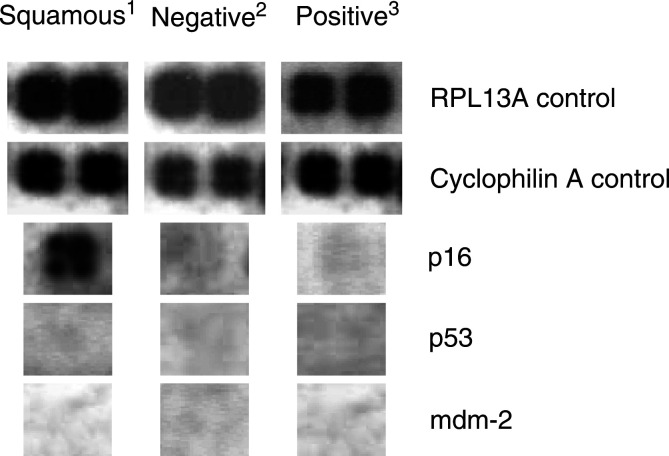
Human p53 Signalling Pathway Gene Array signals obtained for a selection of genes when hybridised with RNA extracted from: 1=oesophageal squamous epithelial biopsies; 2=p53 immunonegative; 3=p53 immunopositive paraffin-embedded oesophageal adenocarcinoma sections.

The arrays employed harboured two spots for each of five constitutively expressed control genes, which displayed strong hybridisation signals on all arrays utilised. Hence, validating the adequate quality of RNA extracted from both fresh and archival material. Figure 2

displays two examples: the RPL13A and cyclophilin A control genes.

When comparing gene expression patterns in the adenocarcinomas (both immunonegative and immunopositive) to the normal squamous tissues, the most striking difference observed was the loss of p16 gene expression in both cancer arrays (Figure 2). A slight downregulation of the MAP2K and BBC3 genes was also noted in both the adenocarcinoma arrays when compared to the normal squamous RNA gene expression pattern. However, due to the limited amount of RNA available for the cancers, these preliminary observations could not be quantitated.

For the genes examined on the p53 array, there were no distinct differences in the gene expression patterns between the immunopositive *vs* immunonegative oesophageal adenocarcinomas. The preliminary data indicated there was a slight downregulation of the HSP70 and GADD45A genes; however, no further RNA was available to verify this observation by quantitative PCR. It was also notable that no increase in expression of the p53 and mdm-2 genes was seen in the immunopositive samples, despite the presence of strong p53 protein accumulation (Figure 2).

## DISCUSSION

The reported frequencies of p53 gene mutations, deletions and p53 immunopositivity vary quite considerably for oesophageal adenocarcinoma. Consequently, in this investigation the aim was to completely characterise the p53 status in a Welsh population. This was performed at the gene sequence, chromosomal, mRNA and protein levels.

Only 10% of the oesophageal adenocarcinomas examined were found to contain a mutation within the p53 gene. Just one of these was identified within a hotspot (codon 245), while those remaining were detected directly adjacent to the hotspot codons. The mutation frequency determined in this investigation was considerably lower than reported in the general literature; nevertheless, two other studies documented comparably lower p53 mutation frequencies of 8 and 12% (also at codon 245) (Casson *et al*, 1991; Gonzalez *et al*, 1997). It is unlikely that just examining exons 5–8 missed many mutations, as >90% of the mutations documented in a wide range of tumours are found within this region (Hamelin *et al*, 1994; www.iarc.fr). This discrepancy could be due to several factors. Differing methodologies may be responsible, for example, PCR-based techniques alone may introduce artefacts (due to errors introduced by the polymerase enzyme) if they are not confirmed by a second independent experiment. Additionally, RFLP and sequencing will only identify mutations if they are present in over 10% of cells within the tumour. If mutations were present in a very small subclone of cells, then they may not have been detected with the techniques employed in this study. Hence, investigations using more sensitive molecular techniques may result in the identification of rare p53 mutations (Jenkins *et al*, 2003). There are also several investigations that have used immunohistochemistry as a direct indicator of p53 gene mutations (Hardwick *et al*, 1994; Rioux-Leclercq *et al*, 1999; Younes *et al*, 2000), but it can be clearly seen by the results in this and other studies that p53 mutations alone are not responsible for abnormal accumulation of the protein (Battifora, 1994; Coggi *et al*, 1997; Soslow *et al*, 1999). Differing sample sizes and types (i.e. fresh tissues *vs* paraffin-embedded archival samples) may cause variability, as would different populations and their habits (e.g. ethnicity/diets/proportion of smokers/alcohol intake). Finally, variation in the statistical methods applied may provide another source of discord.

Several investigations have focused on the frequency of p53 allelic loss in oesophageal adenocarcinoma, with LOH analysis (Gleeson *et al*, 1998; Morgan *et al*, 1998; Dolan *et al*, 1999). This technique involves the detection of gene dosage imbalances by PCR-based microsatellite genotyping; however, there are a number of methodological difficulties (Tomlinson *et al*, 2002). Tumour samples not only contain contaminating normal cells, but are also genetically heterogeneous; therefore, a deletion would have to be present in a substantial number of cells to be detected by LOH analysis. In addition, most tumours are aneuploid and considerable LOH frequency differences have been detected between flow-sorted aneuploid and diploid cells (Bonsing *et al*, 2000). LOH-based studies therefore tend to be inconsistent, particularly as definitions for LOH thresholds are variable (Tomlinson *et al*, 2002). In contrast, FISH involves direct visualisation of the nuclear DNA content on a single-cell basis. The technique is not as high throughput as LOH analysis, but it is more sensitive and can provide additional information, such as the actual position and proportion of cells with allelic deletions within the tumour. FISH was therefore the technique of choice in the present study.

Allelic loss of p53 was consequently detected in 24% of oesophageal adenocarcinomas, but in each of these samples only 14–24% of cells displayed the aberration. As tumours are clonally derived (Nowell, 1976), it would be expected that an abnormality responsible for expansion of the neoplasm would be present throughout – this was not observed in these samples. However, the cells with loss of one p53 allele were always clustered, suggesting they were the result of a subclone that had arisen within the tumours during development and thus did not arise from the original progenitor cells. If we had performed LOH analysis, the clustered pattern of p53 loss would never have been identified, and it is likely that p53 deletions would not have been detected in any of our samples, as at least 75% of cells would have retained both alleles. The threshold that defines significant signal loss may therefore not have been reached. Of additional interest was the fact that p53 gene mutations and deletions did not coexist within any of the samples examined, further suggesting complete p53 inactivation is not an important event in the development of oesophageal adenocarcinoma.

The most important finding in this investigation was that while 83% (24 out of 29) of the adenocarcinomas were immunopositive for p53 protein, only 9.6% (three out of 31) contained a gene mutation. The remaining 21 cases with p53 immunoreactivity all had a normal p53 sequence between exons 5–8. It is possible that mutations may be present in the exons that were not evaluated in this study. However, this would be unlikely to account for the high level of p53 protein positivity detected, as very few mutations have been detected outside of exons 5–8 (www.iarc.fr); therefore, indicating nonmutational p53 protein stabilisation is responsible. A recent publication has demonstrated p53 immunostaining in a variety of normal tissues when using the D07 antibody (Pillai *et al*, 2003), but in the present study several cases were distinctly p53 immunonegative; therefore, suggesting all the immunopositive cases were due to abnormal protein accumulation. Several other causes for p53 protein accumulation exist: (1) overexpression of the p53 gene; (2) stabilisation of p53 via overexpression of mdm-2 or other pathways involved in the control of p53 stability; (3) dysfunctional p53 degradation pathway; (4) abnormal post-transcriptional/translational p53 modifications. To investigate these possibilities, RNA extracted from samples that were immunonegative and those displaying very strong positivity for p53 protein were applied to arrays spotted with genes involved in p53 upstream and downstream signalling pathways. Five RNA samples from each group were pooled, hence genes altered in individual tumours would not have been detected. The overall expression pattern was examined, as this would indicate the common changes present in all the tumours (from the immunopositive and immunonegative groups) that were responsible for accumulation of the p53 protein.

The transcripts examined in the present study were expressed at similar levels in both the p53 protein positive and negative samples; in addition, there was no difference in the expression levels of the p53 and mdm-2 genes (Figure 2). Although this expression analysis did not indicate the possible underlying cause for the protein accumulation, it did rule out overexpression of the p53 and mdm-2 genes as possibilities, indicating some other mechanism must be involved.

The gene expression patterns between the normal squamous epithelium and oesophageal adenocarcinoma were also compared. Expression of the p16 tumour suppressor gene was considerably down regulated; hence, was probably a common event in all of the tumours, which is in agreement with the literature. LOH at the p16 locus and methylation of the genes' promoter region have been frequently identified in both premalignant Barrett's epithelium and oesophageal adenocarcinoma – events that have been strongly associated with loss of p16 expression (Wong *et al*, 1997; Klump *et al*, 1998; Bian *et al*, 2002). In addition, preliminary observations indicated the MAP2K and BBC3 genes were slightly downregulated. Mitogen-activated protein kinase kinase (MAP2K) is a member of the MAP kinase family, and mediates a cellular response to environmental stresses and proinflammatory cytokines through signal transduction cascades. bcl2-binding component 3 (BBC3) is a mediator of p53-associated apoptosis and although its expression in human malignancies is relatively unexplored, suppression might be expected to enhance the survival of neoplastic cells (Han *et al*, 2001). As the RNA samples examined were pooled, it is possible that the expression of these genes were lost in a single case and not in the others, hence resulting in a slight overall reduction. Further gene expression investigations on individual oesophageal adenocarcinomas are therefore warranted.

Paraffin-embedded tissues are an invaluable source of material for molecular investigations. However, reliable extraction of RNA from archival samples is problematic. The most successful RNA extraction method to date was utilised in the present study (Lewis *et al*, 2001), but the yields were low and the RNA was without doubt degraded. Despite the compromised nature of the RNA obtained upon extraction from paraffin-embedded sections, high-quality data has been previously obtained upon hybridisation to microarrays, with quantitative detection of up to 80% of genes when compared to fresh-frozen samples (Lewis *et al*, 2001). However, this is quite controversial and the use of RNA recovered from archival tissue samples for array analysis requires extensive validation. It is therefore important to note that the information obtained in the present gene expression study is preliminary data. Distinct gene expression differences, such as the loss of p16 in the tumour samples demonstrate reliable alterations, but the results are more debatable where only slight differences were seen. Further investigations are thus also required to assess both the frequency and biological significance of the potential gene expression alterations identified here.

To summarise, this investigation has demonstrated that 9.6, 24 and 83% of oesophageal adenocarcinomas contained p53 gene mutations, deletions and p53 protein immunopositivity, respectively. Mutations did not coexist with deletions of the gene and although all cases with a p53 mutation were immunopositive, most immunopositive cases had no demonstrable p53 mutation. Thus, immunohistochemistry is a poor indicator of p53 gene mutations in oesophageal adenocarcinoma. Although overexpression of the mdm-2 and p53 genes can be ruled out as possible causes, the underlying mechanism responsible for the accumulation of the protein has yet to be determined and requires further investigation.
